# Single-cell transcriptome profiling highlights the importance of telocyte, kallikrein genes, and alternative splicing in mouse testes aging

**DOI:** 10.1038/s41598-024-65710-0

**Published:** 2024-06-26

**Authors:** Wuyier Guo, Ziyan Zhang, Jiahui Kang, Yajing Gao, Peipei Qian, Gangcai Xie

**Affiliations:** Institute of Reproductive Medicine, Medical School, Nantong University, Qixiu Road 19, Nantong, 226001 China

**Keywords:** Data mining, Senescence, Transcriptomics

## Abstract

Advancing healthcare for elderly men requires a deeper understanding of testicular aging processes. In this study, we conducted transcriptomic profiling of 43,323 testicular single cells from young and old mice, shedding light on 1032 telocytes—an underexplored testicular cell type in previous research. Our study unveiled 916 age-related differentially expressed genes (age-DEGs), with telocytes emerging as the cell type harboring the highest count of age-DEGs. Of particular interest, four genes (Klk1b21, Klk1b22, Klk1b24, Klk1b27) from the Kallikrein family, specifically expressed in Leydig cells, displayed down-regulation in aged testes. Moreover, cell-type-level splicing analyses unveiled 1838 age-related alternative splicing (AS) events. While we confirmed the presence of more age-DEGs in somatic cells compared to germ cells, unexpectedly, more age-related AS events were identified in germ cells. Further experimental validation highlighted 4930555F03Rik, a non-coding RNA gene exhibiting significant age-related AS changes. Our study represents the first age-related single-cell transcriptomic investigation of testicular telocytes and Kallikrein genes in Leydig cells, as well as the first delineation of cell-type-level AS dynamics during testicular aging in mice.

## Introduction

It has been estimated that approximately 30–50% of infertility cases may stem from male factors, including various influences such as lifestyle choices, exposure to gonadotoxic substances, endocrinopathies, and the aging process itself^1^. Negative impacts of male aging on their reproductive abilities have been observed previously, including a progressive decline in testosterone level attributable to male hypogonadism, deterioration in key semen quality measurements, and an elevated risk of miscarriage in female partners (as reviewed by Eisenberg et al.^1^). The testis serves as the primary organ responsible for male reproduction, generating both sperm and male hormones. Aging-related dysfunction in both the germ and somatic cells of the testis has been observed, encompassing phenomena such as the loss of spermatogonial stem cell (SSC) quiescence, increased gene mutation rates, and an imbalanced redox status in sperm, along with morphological and functional impairments in Leydig and Sertoli cells (reviewed by Shijue Dong et al.^2^). Interestingly, a distinctive aging pattern was observed in human sperm compared to other tissues, such as an increase in telomere length in aged sperm^3^, underscoring the potential of studying aged testes to deepen our understanding of the aging process. While the concentration of circulating testosterone typically fluctuates within the normal range with age for most men, a minority may encounter symptoms of late-onset hypogonadism (LOSH) in their advanced age, including consistently low serum total testosterone levels, erectile dysfunction, diminished morning erections, and even obesity and declining health^4^. Such investigations highlight the importance of examining age-related gene expression changes in the testes, which could offer valuable insights for the healthcare management of elderly men.

With the advent of single-cell sequencing technologies^5,6^, numerous studies have investigated the single-cell transcriptomic alterations associated with testis aging^7–9^. Among these endeavors, through single-cell transcriptome profiling of testes from young and old men, Jingtao Guo and colleagues revealed transcriptional changes in various cell types of the human testis, including metabolic signaling alterations in Sertoli cells, hedgehog signaling changes in Leydig cells, and increased inflammation in multiple cell populations^8^. Intriguingly, they also revealed an age-related correlation between testis dysregulation and body mass index. Similarly, another study^7^ focused on murine testes aging, utilizing single-cell RNA sequencing to uncover a surge in inflammation within macrophage and endothelial cells, coupled with the down-regulation of genes involved in DNA repair and telomere maintenance across Sertoli, Leydig, and germ cells. Additionally, a separate exploration delved into the single-nucleus transcriptome profiling of testes from young and aged crab-eating monkeys^9^, highlighting a waning presence of spermatogonial stem cells, compromised spermatogenesis, and a repertoire of differentially expressed genes in somatic cells. Their study underscored the importance of WT1 gene expression in Sertoli cell senescence and elucidated age-associated microenvironment changes impacting germ cells. Notably, however, certain testicular cell types, such as telocytes, have remained overlooked in these studies. Moreover, none have explored the ramifications of alternative splicing on testes aging.

Telocytes represent a distinct type of interstitial cell characterized by their unique cell morphology, featuring exceptionally long and slender prolongations known as telopodes (Tps). Telocytes were recently re-discovered and the term was coined by Popescu and colleagues^10^, exhibiting Tps with lengths of approximately 1000 μm and thicknesses between 0.05 and 0.2 μm^11^. These Tps display a distinctive moniliform structure, comprising alternating narrow segments termed podomers and dilated regions referred to as podoms. Telocytes have been widely found in different tissues, including gut, urinary system, cardiovascular system, skin, and skeletal muscle, as reviewed^12^ by Yuhua Zhang and Hu Tian. Telocytes have been demonstrated to express CD34 and PDGFRA (PDGFRα)^11,12^, and in one investigation, the labeling of PDGFRα was utilized for the identification of telocytes within gut tissue^11^. Notably, telocytes were also recently found in the testes across multiple species, including humans^13^, mice^14^, and rats^15^. These cells were observed to localize proximally to peritubular myoid cells (PMCs), enveloping seminiferous tubules^13–15^. A study employing single-cell RNA sequencing (scRNA-seq) of mouse testes also noted the identification of telocytes^16^. However, to the best of our knowledge, investigations into telocytes in the testes during the aging process remain absent from the existing literature.

Moreover, alternative splicing has been implicated in cell senescence, organism aging, and longevity, as reviewed in previous studies^17–19^. It has been claimed that about 30% of alternative splicing events occur during the aging process, impacting genes involved in neuronal function in the human brain and age-associated brain disorders, as discussed by Baralle and Romano^18^. One notorious example is the premature aging disease Hutchinson-Gilford progeria syndrome (HGPS), which is caused by mutations affecting LMNA splicing patterns^20,21^. The significance of alternative splicing extends to germ cell development, where disruption of splicing regulators has been shown to impair meiosis and lead to male infertility^22^. Bulk RNA-seq analyses have revealed associations between alternative splicing regulation and spermatogonial stem cells (SSCs) aging and differentiation, identifying dysregulation of several splicing regulators during aging^23^. However, while previous single-cell transcriptome profiling of aged testis^7^ suggested a potential link to alternative splicing based on gene enrichment analysis, comprehensive genome-wide profiling of splicing events at the single-cell level during testis aging process remains unexplored.

In this study, we conducted a comprehensive analysis of transcriptome profiles at the single-cell level, encompassing over 40,000 individual mouse testicular cells from both old and young mice. Our study explored age-related transcriptomic changes in testis telocytes and examined alterations in global alternative splicing (AS) events across all testicular cell types. Unexpectedly, our findings revealed that telocytes exhibited the most pronounced changes in gene expression level during testicular aging, and hundreds of AS events were found to be associated with aging in the testis. Remarkably, despite the prevalent pattern of age-related differentially expressed genes (DEGs) observed in somatic cells, we noted a contrasting trend in AS events, with a higher prevalence of age-related AS alterations occurring in germ cells compared to somatic cells. Furthermore, we identified a cluster of kallikrein genes that were specifically downregulated in aging testicular Leydig cells, which might provide valuable insights into potential mechanisms underlying Leydig cell aging. Collectively, our studies emphasize the importance of telocytes and alternative splicing in the aging process of the testes and underscore the contribution of Leydig Kallikrein genes to testicular aging. Our discoveries open new avenues for understanding the mechanisms of testicular aging, offering novel targets for potential treatments.

## Results

### Single-cell transcriptome profiling of young and old mouse testes

**Figure 1:**
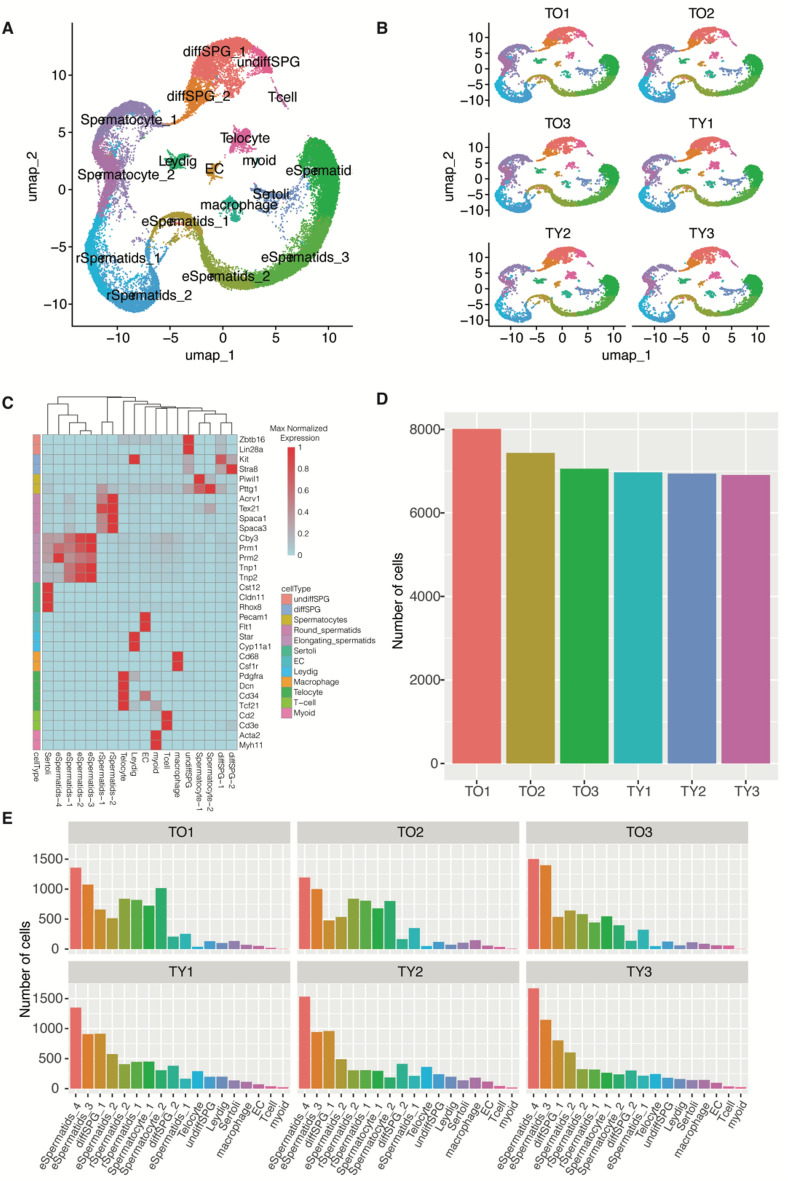
Single-cell transcriptome study of mouse testes aging. (**A**, **B**) UMAP (Uniform Manifold Approximation and Projection) representation of different cell types of mouse testes. For all samples merged (**A**) or for each sample individually (**B**) (TO: old sample, TY: young sample, EC: Endothelial Cell, undiffSPG: undifferentiated spermatogonia, diffSPG: differentiated spermatogonia, rSpermatids: round Spermatids, eSpermatids: elongating Spermatids). (**C**) Expression of known marker genes for each cell type. For each gene, the cell-type level average expression values were further normalized by the maximum average expression value of all cell types. (**D**) Number of cells analyzed for each sample. (**E**) Distribution of the number of cells for each cell type in each sample. The arrangement of cell type names in the barplot is based on their total number of cells in descending order.

To investigate transcriptome alterations at the single-cell level in aged mouse testes, we conducted an analysis on testicular samples obtained from three young (3 months old, sample name prefixed with TY) and three old mice (23 months old, sample name prefixed with TO) using single-cell RNA sequencing technology (scRNA-seq). We employed the Singleron CLindex sample multiplexing method to barcode and pool all samples during preparation and sequencing stages of scRNA-seq (Sample details provided in Table S1). Utilizing this multiplexing approach, we ensured consistent sequencing quality across all six samples, as evidenced by various metrics such as the total number of unique molecular identifiers (UMIs) per cell, the number of genes detected per cell, and the percentage of transcripts originating from the mitochondrial genome in each cell (Figure S1). In total, we acquired the transcriptome profiles from 43,323 mouse testicular cells, encompassing 18 distinct cell types, including 7 somatic cells and 11 germ cells representing different stages of spermatogenesis (Fig. 1A). Notably, all identified cell types were present in both young and old mice testes (Fig. 1B).

The annotation of cell types was performed using established testis marker genes from previous studies, including Star and Cyp11a1 for Leydig cells; Cst12, Cldn11, and Rhox8 for Sertoli cells; Zbtb16 and Lin28a for undifferentiated spermatogonia (undiffSPG); Kit and Stra8 for differentiated spermatogonia (diffSPG_1,diffSPG_2); Pecam1 and Flt1 for endothelial cells (EC); Cd68 and Csf1r for macrophages; Prm1 and Prm1 for elongating spermatids (eSpermatids_1,eSpermatids_2, eSpermatids_3, eSpermatids_4); Spaca1, Spaca3, Tex21 and Acrv1 for round spermatids (rSpermatids_1, rSpermatids_2); Piwil1 and Pttg1 for spermatocytes (Spermatocyte_1, Spermatocyte_2). The expression levels of known marker are depicted in Fig. 1C, and the sources of known marker genes are list in Table S2. On average, more than 1000 marker genes were identified for each cell type based on Seurat analysis (Table S3). A comparable number of cells were profiled for each sample (Fig. 1D), with 22, 504 cells from aged mouse testes and 20, 819 cells from the young ones. Germ cells predominated, comprising a total of 39, 228 cells, including 993 undiffSPGs and 5955 diffSPGs (Fig. 1E).

Interestingly, we identified 1032 cells as telocytes (Figs. 1A,E), characterized by higher expression of Cd34, Pdgfra, Dcn, and Tcf21 (Fig. 1C). Noticeably, many more telocytes were identified in this study than myoid cells (Fig. 1A). The telocytes identified in this study can be distinguished from myoid cells based on known marker genes (Figure S2A). Specifically, myoid marker genes Acta2 and Myh11 were scarcely detectable in the telocytes, highlighting their distinct genetic profile (Figure S2A). To address the possibility of telocyte misidentification, we conducted clustering analysis on the telocyte population to elucidate its subpopulations. Four distinct telocyte subpopulations were delineated (T1, T2, T3, T4) (Figure S2B), each demonstrating expression of telocyte marker genes (Cd34, Pdgfra, Tcf21, Dcn), while exhibiting minimal expression of myoid marker genes (Acta2, Myh11) (Figure S2C). Furthermore, the four telocyte subpopulations can be differentiated based on the identified marker genes (Figure S2D, Table S4).

### Age-related gene expression and cellular composition changes

**Figure 2:**
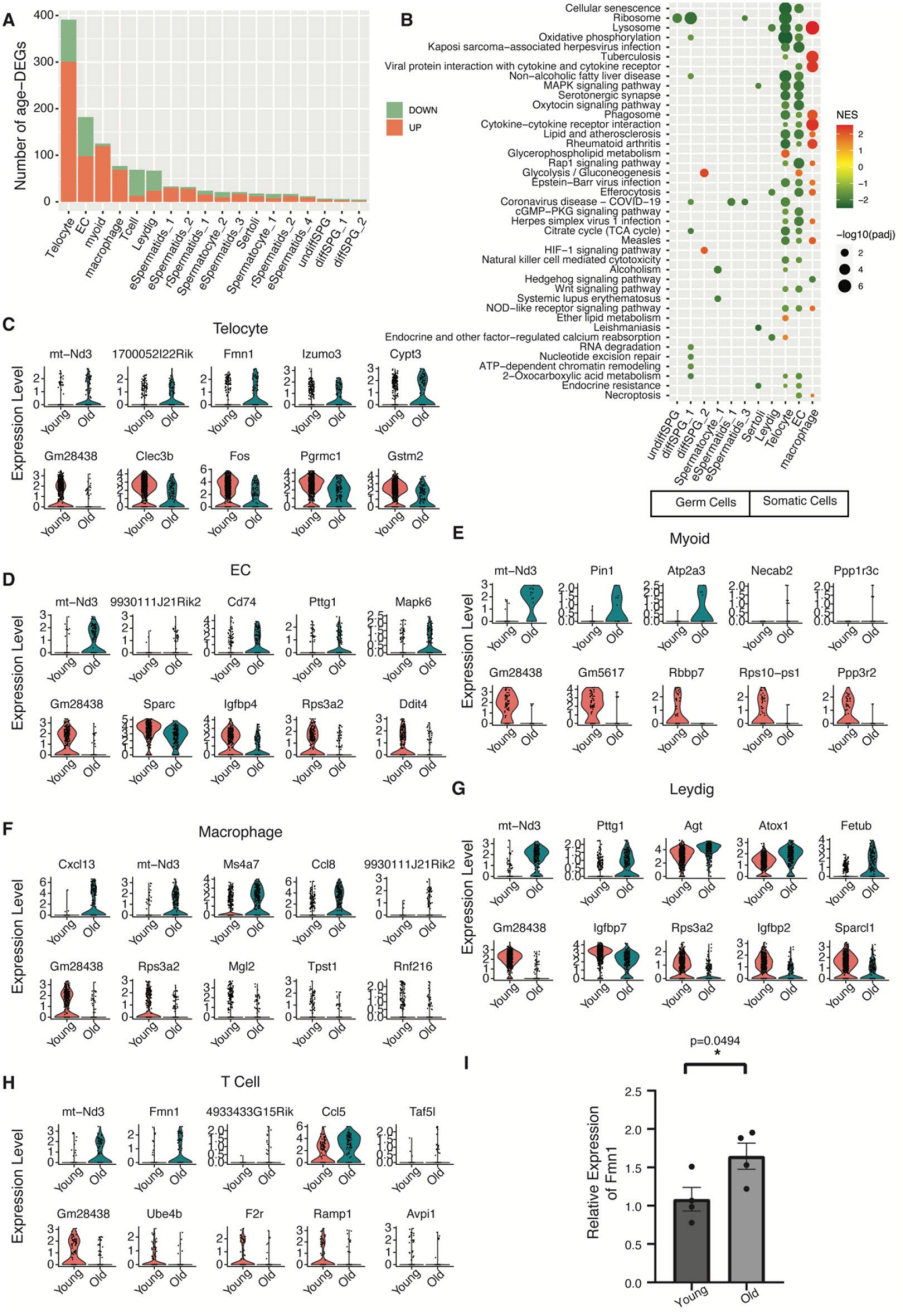
Cell-type-level gene expression changes in aging mouse testes. (**A**) Distribution of the number of age-related differentially expressed genes (age-DEG) for each cell type of mouse testis. (**B**) Gene Set Enrichment Analysis (GSEA) of age-DEGs (NES: Normalized Enrichment Score, padj: adjusted *P*-value). (**C**–**H**) Violin-plot for the top-five up-regulated and down-regulated age-DEGs in telocyte (**C**), EC (**D**), myoid (**E**), macrophage (**F**), Leydig (**G**) and T cell (**H**). (**I**) RT-qPCR validation of the expression change of Fmn1. Bar plot data is mean $\pm$ SEM, *P-*values were calculated using two-sided Student’s unpaired *t*-tests, *P < 0.05 , Young mice (n = 4), Old mice (n = 4).

By comparing the gene expression profiles in the testes of old and young mice, we identified 916 age-related differentially expressed genes (age-DEGs) (Table S5). Noteworthy is the prevalence of age-DEGs in telocytes, including 301 up-regulated and 90 down-regulated age-DEGs (Fig. 2A). The top six testicular cell types with highest number of age-DEGs were exclusively from somatic cells, ranked in the order of telocytes, EC, myoid cells, macrophages, T cells, and Leydig cells (Fig. 2A). Intriguingly, spermatogonia (SPG), both undifferentiated (undiffSPG) and differentiated (diffSPG_1, diffSPG_2), manifested the least number of age-DEGs (Fig. 2A).

Next, based on the genes ranked by the fold change in expression levels between old and young mice testes, we conducted gene set enrichment analysis (GSEA) for each cell type utilizing the KEGG pathway database (Fig. 2B, Table S6). In the macrophages of aged testes, genes related to lysosome and phagosome functions exhibited upregulation, whereas those linked with the MAPK signaling pathway, oxytocin signaling pathway, and cGMP-PKG signaling pathway were down-regulated in EC and telocytes (Fig. 2B). Furthermore, in Leydig cells from aged testes, genes related to “endocrine and other factor-regulated calcium reabsorption” were down-regulated (Fig. 2B), including several Kallikrein genes (see next section for detailed examination).

Figure 2C–H present the top five up-regulated or down-regulated age-DEGs for the six testicular cell types with the highest number of age-DEGs (full list in Table S5). Among these top age-DEGs, the mitochondrial gene mt-Nd3, involved in mitochondrial electron transport, was consistently upregulated across six somatic cell types. Subsequent examination revealed significant upregulation of mt-Nd3 in 13 testicular cell populations of aged testes (Table S5). Moreover, certain non-coding RNA genes were also prominent among the top age-DEGs (Fig. 2C–H, Table S5), including Gm28438, which was down-regulated in 14 cell types of aged testes (Table S5). Another age-DEG showing widespread age-related transcriptional dysregulation was formin 1 (Fmn1), found to be up-regulated in 13 testicular cell populations of aged testes (Table S5). Formins belong to one of the three known classes of actin nucleators (proteins that promote actin filament formation)^24^ and FMN1 has been found to be related to cancer motility and mechanical cohesion formation^25^. In this study, we observed upregulation of Fmn1 in aged mice testes, and its transcriptional changes were further validated by qRT-PCR experiments (Fig. 2I).

Furthermore, we identified notable changes in cell population proportions within the aged testis based on our scRNA-seq datasets. As outlined in Table S7, we observed significant increases in the proportions of myoid cells, Leydig cells, telocytes, and differentiated SPGs in the aged testis. Conversely, the proportions of round spermatids (rSpermatids_2) and spermatocytes (Spermatocyte_1) were significantly decreased (two-sided t-test *P-*value < 0.05). These findings suggest a shift in cell populations between young and aged testes due to aging, highlighting the necessity for further experimental validation.

### Leydig-specific down-regulation of kallikrein genes

**Figure 3:**
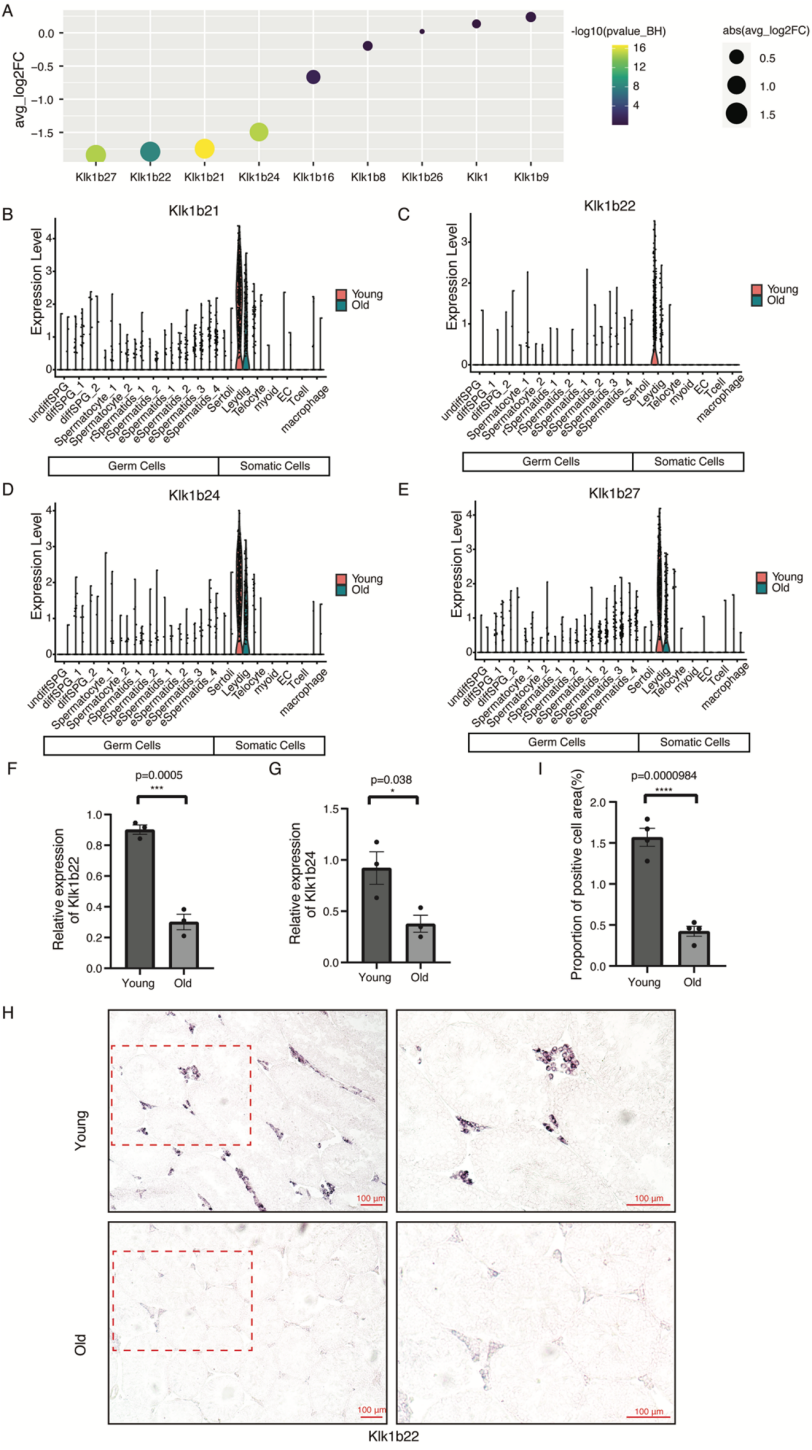
Age-related down-regulation of kallikrein genes in Leydig cells of mouse testes. (**A**) Age-related gene expression changes (old vs young) for kallikrein genes in mouse testis (*P* value_BH: BH adjusted *P*-value, abs(avg_log2FC): absolute value of log2-transformed fold-change of the average expression between old and young groups). (**B**–**E**) Violin-plot for the four kallikrein genes that were significantly down-regulated in aged mouse testis. Klk1b21 (**B**), Klk1b22 (**C**), Klk1b24 (**D**), and Klk1b27 (**E**). (**F**, **G**) RT-qPCR validation of the expression of Klk1b22 (**F**) and Klk1b24 (**G**). (**H**) RNA in situ hybridization examination of Klk1b22 in the young (3 months) and old (23 months) mouse testes. (**I**) Comparison of the proportion of Klk1b22 staining positive area in the old and young mice testes. Bar plot data is mean $\pm$ SEM, *P-*values were calculated using two-sided Student’s unpaired *t*-tests, *P < 0.05, ***P < 0.001, ****P < 0.0001, Young mice (n = 3 for **F**, **G**; n = 4 for I), Old mice (n = 3 for **F**, **G**; n = 4 for I).

Kallikreins belong to a subgroup of serine proteases, and are pivotal enzymes regulating various biological processes, including hormone activities, growth factor modulation, extracellular matrix organization, and membrane receptor functions^26^. There are more than 35 kallikrein genes in the mouse genome, which form gene clusters and are colocalized in the genome^27^. In our investigation, we identified a total of 28 kallikrein genes in mouse testes, some of which were specifically expressed in Leydig cells (Figure S3). Notably, we identified four kallikrein genes exhibiting specific down-regulation within Leydig cells of aged mouse testes (Fig. 3A, Table S5), namely Klk1b21 (Fig. 3B), Klk1b22 (Fig. 3C), Klk1b24 (Fig. 3D), and Klk1b27 (Fig. 3E). Subsequent RT-qPCR validated the down-regulation of Klk1b22 (Fig. 3F) and Klk1b24 (Fig. 3G) in mouse testes. Furthermore, RNA in situ hybridization (ISH) targeting Klk1b22 transcripts confirmed a significant (*P* = 0.0000984) decrease in expression within Leydig cells of aged mouse testes (Fig. 3H,I), with no detectable staining observed in negative control experiments utilizing sense probes for the Klk1b22 gene (Figure S4). A previous study identified Klk1b24 as exclusively expressed in the Leydig cells of adult mouse testes, with transcripts detection initiating at 4 weeks of age^28^. Herein, we not only reaffirm the Leydig-specific expression of Klk1b24 but also identify three additional Leydig-specific kallikrein genes: Klk1b21, Klk1b22, and Klk1b27 (Figures B–E). Crucially, all identified Leydig-specific kallikrein genes were significantly down-regulated in the testes of aged mice.

### Age-related changes in mouse testicular telocytes

**Figure 4:**
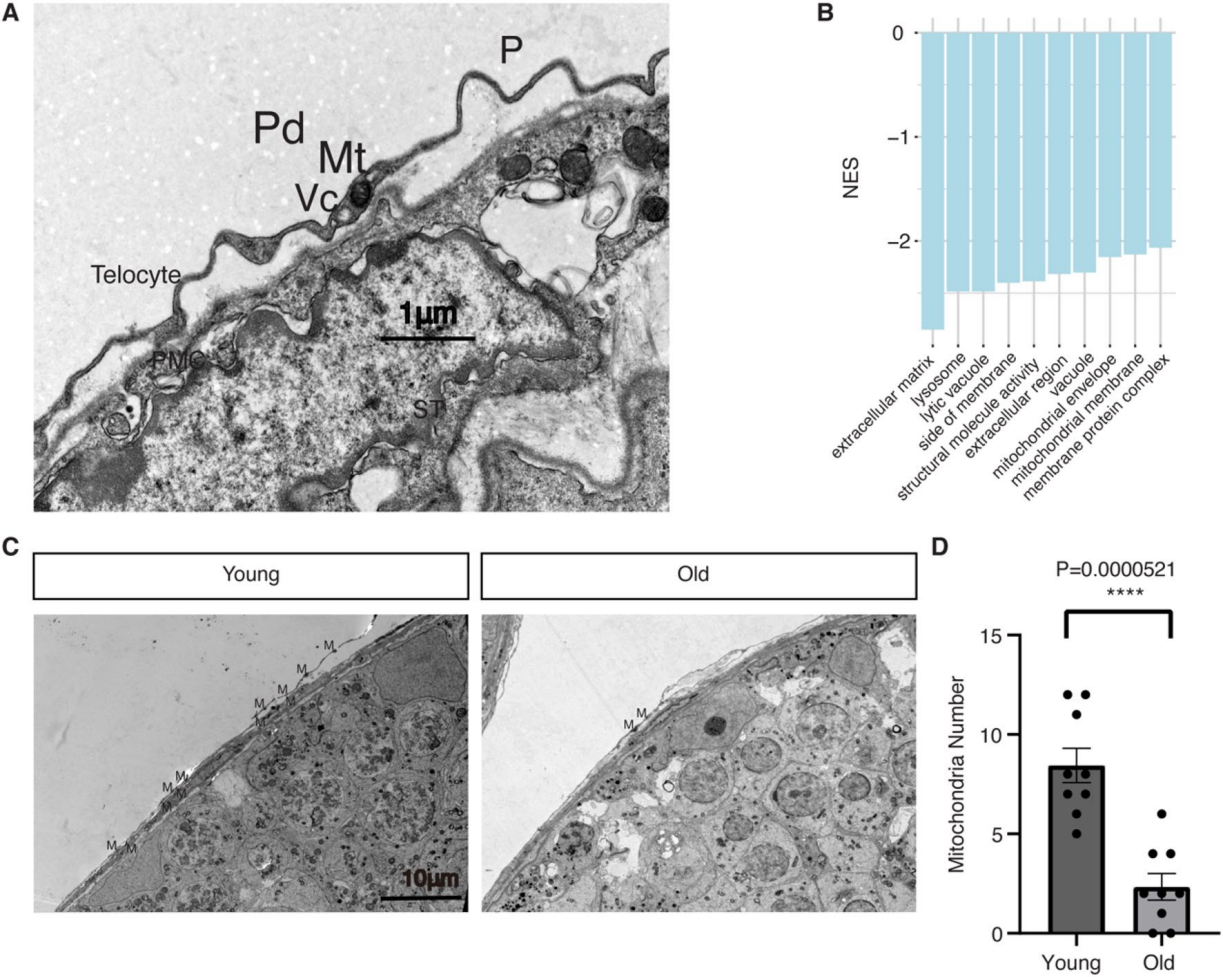
Age-related changes in the telocytes of mouse testes. (**A**) TEM structure of the telocyte in mouse testis. PMC: peritubular myoid cell; ST: seminiferous tubule; Vc: Vesicle; Mt: Mitochondria; Pd: Podom; P: Podomer. Scale Bar = 1μm. TEM: Transmission Electron Microscope. (**B**) GO enrichment analysis of telocyte age-DEGs. NES values were the normalized enrichment scores calculated by GSEA method. (**C**) Mitochondrial reduction in testicular telocyte of aging mice. M: Mitochondria. Scale Bar = 10μm. (**D**) Barplot illustration the comparison of the number of mitochondria between old and young mice. Error bars represent the SEM. ****P < 0.0001, Nine fields of TEM view for each age group. The number of mitochondria in the telocyte was normalized to standard length (20μm) of telopode (Pd + P).

Telocytes exhibit distinctive morphologies, characterized by their remarkably elongated and slender telopodes (Tps)^11^. In this study, we used transmission electron microscopy (TEM) to examine the structure of telocytes in mouse testes, confirming this structure with recurrent podoms (Pd) and podomers (P) (Fig. 4A). Telocytes are typically located at the outermost layer of seminiferous tubules, near the peritubular myoid cells (PMCs) (Fig. 4A).

Our study revealed 391 age-DEGs within telocytes (Table S5). Intriguingly, based on Gene Set Enrichment Analysis (GSEA), we found an enrichment of down-regulated genes associated with the Gene Ontology (GO) terms “mitochondrial envelope” and “mitochondrial membrane” (Fig. 4B). Next, we examined all the mitochondrial genes detected in the telocytes and found that eight of them were expressed at relatively higher levels compared to the other mitochondrial genes (Figure S5A). The average expression of all detected mitochondrial genes was lower in the telocytes of old mice compared to those of young mice (Figure S5B). Specifically, the average expression levels of each of the eight highly expressed mitochondrial genes were down-regulated in the telocytes of old mice compared to those of young mice (Figure S5C). Among these, six mitochondrial genes (mt-Cytb, mt-Nd1, mt-Co1, mt-Nd4, mt-Nd5, and mt-Nd2) showed statistically significant down-regulation (*p* < 0.05) without multiple testing correction. One gene (mt-Nd5) remained significant after multiple testing correction (adjusted *p* < 0.05) (Table S8). Finally, comparing the TEM structures of telocytes in the testes of old and young mice revealed a significant reduction in the number of mitochondria in aged mice, consistent with the observed mitochondrial gene expression changes (Fig. 4C,D).

GSEA also revealed an enrichment of age-related down-regulation in genes associated with the GO terms “extracellular matrix” and “side of membrane” in telocytes (Fig. 4B). The down-regulation of these genes may impair telocyte cellular contact with other testicular cells. To investigate this hypothesis, we performed immunofluorescence co-staining of the telocyte marker gene, CD34, and the PMC marker gene, ACTA2 (Figure S6). Remarkably, we observed a significantly higher incidence of ruptures in the seminiferous tubules (ST) of aged mice testes, possibly indicating a telocyte-associated deterioration of the ST microenvironment in aging testes.

### Age-related alternative splicing changes

**Figure 5:**
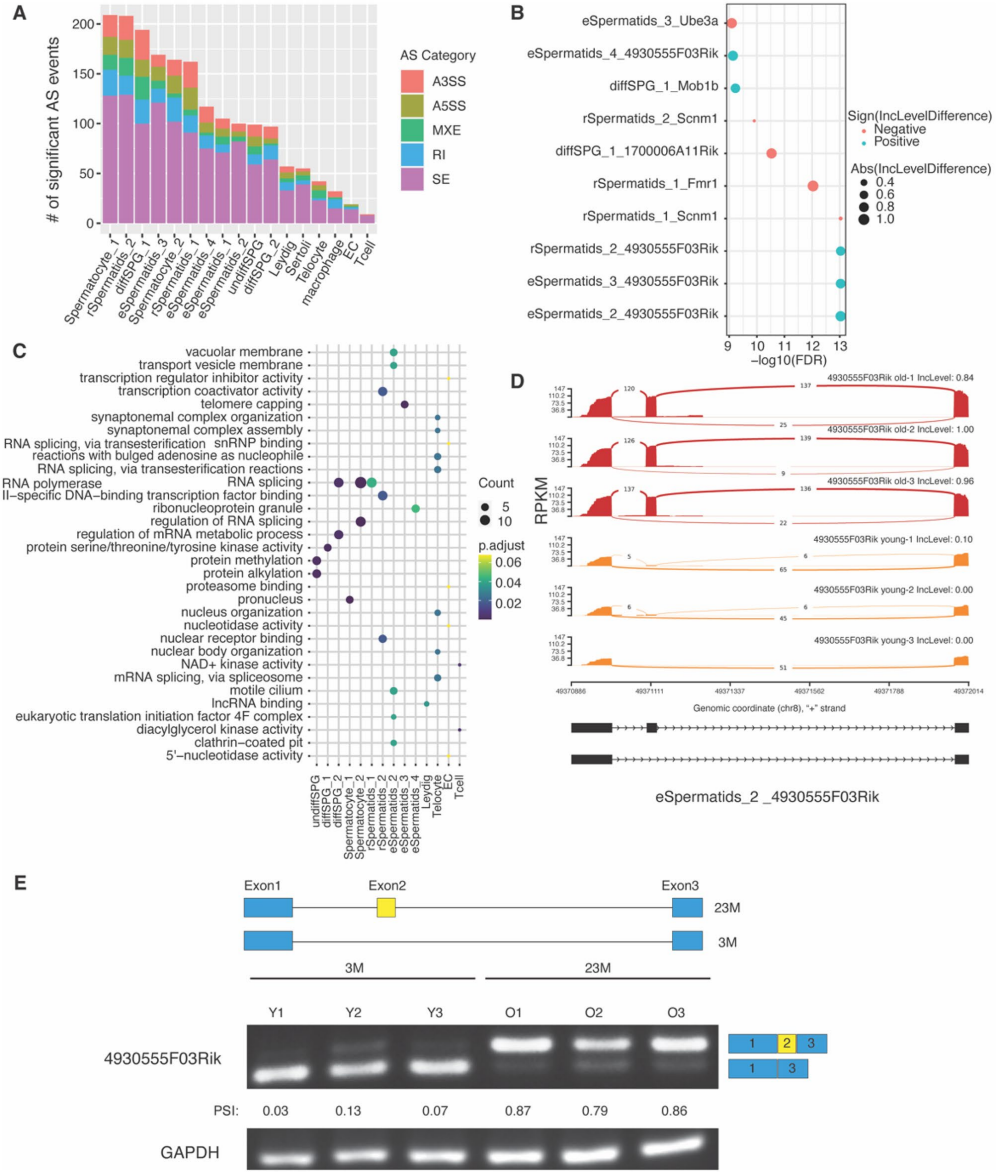
Gene alternative splicing changes in aging mouse testes. (**A**) Distribution of the number of alternative splicing (AS) events observed in aged mice testes. Five categories of AS events were shown. A3SS: alternative 3’ splice site; A5SS: alternative 5’ splice site; MXE: mutually exclusive exons; RI: retained intron; SE: skipped exon. (**B**) Dotplot of the top-10 AS events in aged mice testes (IncLevelDifference: Difference of the inclusion levels between old and young groups). (**C**) Top Gene Ontology (GO) terms enriched with age-related AS genes. (**D**) Sashimi plot of the AS events for 4930555F03Rik gene. The first three exons of 4930555F03Rik were illustrated, and the inclusion level (IncLevel) values for each sample were shown. (**E**) Agarose gel electrophoresis validation of the RT-PCR products of 4930555F03Rik transcripts in the young and old mouse testes. The length of the PCR products was 114bp (base pair) and 84bp, respectively. Three biological replicates for each age group used in the experiments. PSI: Percent Spliced In, Loading control: GAPDH. PSI values were calculated based on the band intensities of RT-PCR products. The raw gel images can be found in Figure S7.

Next, we examined the alternative splicing changes within each cell type of aged mice testes. For each cell type, five alternative splicing events were calculated by rMATS^29^, including skipped exon (SE), alternative 5’ splice site (A5SS), alternative 3’ splice site (A3SS), mutually exclusive exons (MXE), and retained intron (RI). In total, we identified 1838 age-related alternative splicing (age-AS) events associated with 1148 genes in mice testes, including 1154 SE (Table S9), 162 A5SS (Table S10), 208 A3SS (Table S11), 120 MXE (Table S12) and 194 RI (Table S13). Interestingly, most age-AS events occurred in germ cells, with the top five cell types with the highest abundance of age-AS events identified as Spermatocyte_1, rSpermatids_2, diffSPG_1, eSpermatids_3, and Spermatocyte_2 (Fig. 5A). Within each cell type, SE emerged as the primary AS event showcasing the most significant changes in aged-mice testes (Fig. 5A). The top 10 age-AS events were associated with five genes: 4930555F03Rik, Scnm1, Fmr1, Mob1b, and Ube3a (Fig. 5B). Interestingly, the genes associated with age-AS events were enriched in the GO terms “RNA splicing”, “regulation of RNA splicing”, and “snRNP binding” (Fig. 5C), which indicates aging-related dysregulation of alternative splicing of the genes related to the splicing machinery. Besides, age-AS genes were also enriched in “lncRNA binding”, “telomere capping”, “motile cilium”, “protein methylation” and “protein alkylation” (Fig. 5C). Our study revealed many non-coding RNA (ncRNA) genes that exhibited significant AS changes in aged-mouse testes, including 4930555F03Rik, 1700022E09Rik, Gm32828, Gm12637, and 1700010J16Rik (Table S9). The most significant AS event was the SE change of the second exon of the non-coding RNA gene 4930555F03Rik in spermatids (Fig. 5D), which was also further experimentally validated (Fig. 5E).

## Discussion

In previous studies on testes aging^7–9^ utilizing single-cell transcriptome analyses, single-cell RNA sequencing was conducted on each sample separately. However, in our study, we leveraged the advantage of sample multiplexing technology (see the METHODS section), enabling the single-cell RNA sequencing (scRNA-seq) of young and old testes samples within a single pool. This approach substantially mitigates the batch effects inherent in profiling single-cell transcriptomes separately for each sample. Our analysis yielded single-cell transcriptome profiles with a remarkably balanced distribution across different samples (Fig. 1D). Importantly, our investigation led to the identification of over 1000 telocytes in the mouse testes, affording us the unique opportunity to investigate their transcriptome changes during the aging process for the first time. Un-expectedly, we identified more age-related differentially expressed genes (age-DEGs) in telocytes than in other testicular cell types (Fig. 2A), underscoring the significance of studying this specific cell population in the context of testis aging research. Despite a limited number of studies focusing on telocytes in the testis^13–15^, predominantly centered on anatomical and morphological investigations, there exists a notable gap in understanding the transcriptome changes of telocytes in the testes during aging. Telocytes, characterized by their extremely long telopodes, are proposed to interact and communicate with surrounding cells (reviewed by Vannucchi^11^). Based on the observation of more ruptures in the aged testis seminiferous tubules (ST), our study suggests telocyte-associated damage to the ST microenvironment in aging testes. To exclude the possible histological artifacts, we conducted immunofluorescence co-staining experiments on both of cryopreservation tissue and paraffin-wax embedded tissue. Although both young and old tissues underwent the same experimental procedures, more ruptures occurred in the old tissues. However, aside from the damaged ST microenvironment in the old mouse testes, our experiments cannot exclude other possibilities, such as the likelihood that the old tissues are more susceptible to damage in immunofluorescence experiments.

In our analysis of mitochondrial-related changes in the testicular telocytes of old mice, we observed consistent alterations in both global mitochondrial gene expression and the number of mitochondria (Figures S5 and 4C-D). All majorly expressed mitochondrial genes showed down-regulation in testicular telocytes of old mice compared to young ones (Figure S5C), which was consistent with reduced mitochondria in telocytes of old mice testes based on TEM experiments (Fig. 4C,D). However, we also observed that a less abundantly expressed mitochondrial gene named mt-Nd3 showed significantly higher expression level in testicular telocytes of old mice compared to young mice (Figure S5A, Table S8). This observation suggests a complex dysregulation of mitochondrial genes associated with aging. While majorly expressed mitochondrial genes showed aging-related downregulation, a few less abundantly expressed mitochondrial genes might exhibit opposite changes.

Moreover, our investigation unveiled four members of Kallikrein gene family that displayed specific down-regulation within the Leydig cells of aging mice testes (Fig. 3). To the best of our knowledge, there have been no studies about the relationship between Kallikrein genes and testes aging. Leydig cells, located in the interstitial space of seminiferous tubules, play a pivotal role in male physiology by secreting testosterone and other androgens. As males age, there is a gradual decrease in serum testosterone (T) levels, which could further lead to the changes in muscle strength, sexual and cognitive functions, and mood^30^. The aging process of Leydig cells is one of the main factors contributing to the decline in T levels among aged animals^30^. Kallikrein genes, showcasing exceptional responsiveness to steroids and various hormones^27^, coalesce into clustered formations within the genome. Three (Klk1b21, Klk1b24, Klk1b27) of the four age-related down-regulated Kallikrein genes identified in this study, which exhibit Leydig cell-specific expression, were also found to be down-regulated in the testes of androgen receptor mutant mice^31^. The decrease in testosterone secretion observed in aging male mice could potentially contribute to the downregulation of these Leydig-specific Kallikrein genes. Our findings emphasize the necessity for further investigations in the future to elucidate the role of Kallikrein genes in the aging process of Leydig cells.

In contrast to previous single-cell transcriptome studies that predominantly focused on changes in gene expression levels during testes aging^7–9^, this study pioneers the exploration of alternative splicing changes at the cell-type level, providing a novel perspective on the aging process within the testis. In this study, leveraging scRNA-seq clustering results, we generated pseudo-bulk RNA-seq datasets for individual cell types in mouse testes. This approach empowered us to compute cell-type-level alternative splicing changes (AS) using tools traditionally developed for bulk RNA sequencing. Interestingly, many non-coding RNA (ncRNA) genes have been identified as age-related AS genes in this study. Previous studies have elucidated that abnormal expression of long non-coding RNAs (lncRNAs) can detrimentally impact spermatogenesis. For instance, male mice deficient in lncRNA *Teshl* produced sperm displaying aberrant morphology^32^, while knockdown of lncRNA *Gm4665* impeded the transition of round spermatids to elongating spermatids^33^. The age-related alternative splicing changes observed in ncRNAs within germ cells in this study may indicate a potential link between ncRNA dysregulation and abnormal spermatogenesis in the aging testis. Notably, our study revealed an opposite pattern in age-related changes for gene expression and AS in somatic and germ cells. While based on gene expression profiles, we identified more age-DEGs in somatic cells than in germ cells (Fig. 2A), our AS study revealed contrasting results (Fig. 5A). In a study involving aging men^8^, it was demonstrated that age-associated dysregulation was more pronounced in somatic cells than in germ cells, aligning with our findings based solely on gene expression. However, our findings based on AS events illustrated that more genes exhibited dysregulated splicing in germ cells than in somatic cells during testes aging.

In summary, our research presents the first comprehensive exploration into age-related gene expression changes in telocytes and alternative splicing (AS) changes across various cell types in mouse testes. Our analysis identifies telocytes as an important player in testicular aging, with the highest abundance of age-DEGs, highlighting their significance for future studies on testicular aging. Furthermore, our findings spotlight a greater prevalence of AS events in germ cells compared to somatic cells in mouse testes, underscoring their pivotal roles in germ cell aging processes. One limitation of our investigation into alternative splicing in testicular aging is the use of short-read single-cell RNA sequencing, which captures reads of 150 base pairs and only the 3’ end of transcripts. This approach hinders the comprehensive capture of full-length isoforms and results in uneven coverage across transcripts. This limitation could be addressed by leveraging recently developed long-read sequencing technologies^34–36^ in future studies.

## Methods

### Animal

Male C57BL/6 mice of various ages were maintained by the Laboratory Animal Center of Nantong University. Animal care and experiments followed the guidelines of Jiangsu Province Animal Care and were approved by the Animal Care and Use Committee of Nantong University (NO. S20220314-007). The study is reported in accordance with ARRIVE guidelines. The young mice used in this study were 3 months old, while the old mice used were 21 months of age or older (Table S1, Table S14, 21M-29M).

### Single-cell RNA sequencing experiments

Testicular samples were dissected from three young (3 months old) and three old (23 months old) mice for single-cell RNA sequencing. Initially, the fresh testicular samples were washed three times with Hanks’ Balanced Salt Solution (HBSS). Subsequently, they were transferred to a centrifuge tube and finely minced into 1–2 mm fragments using sterile scissors. These tissue fragments were digested using 2 ml of GEXSCOPE® Tissue Dissociation Solution (Singleron) at 37 °C in a 15 ml centrifuge tube with continuous agitation. After that, the samples were filtered through 40-micron sterile strainers and transferred into a 50 ml centrifuge tube, which was then centrifuged at 1000 rpm for 5 min. The supernatant after centrifugation was discarded, and the sediment was resuspended in 1 ml of PBS.

To enable the construction of sequencing libraries for single cells from different samples in one pool, the sample information for each cell was labeled with a sample-specific barcode sequence using the CLindex® Sample Multiplexing Kit (Singleron), following the manufacturer’s instructions. The constructed sequencing library was then sequenced using the Illumina NovaSeq 6000 sequencer (paired-end, 150 base pairs).

### Single-cell RNA sequencing data analyses

The sequencing data from scRNA-seq were preprocessed using CeleScope software (version 1.11.0, https://github.com/singleron-RD/CeleScope), which included a series of steps: cell barcode and UMI decoding, adapter sequence removal, sample barcode decoding, reads mapping, and gene expression counting. Both the mouse reference genome and gene annotation files were downloaded from GENCODE (https://www.gencodegenes.org/mouse/release_M25.html, GENCODE Release version M25, Mouse Reference Genome Version GRCm38.p6). Celescope rna cutadapt (based on Cutadapt^37^ version 3.7) was used to find and remove adapter sequences, primers, and other types of unwanted sequences (parameters: “–minimum_length 20 –nextseq_trim 20 –overlap 10 –insert 150”). The sequencing reads were mapped onto the mouse reference genome using celescope rna star (based on STAR^38^_2.6.1a_08-27, parameters: “–outFilterMultimapNmax 1”), and the gene expression was calculated by celescope rna featureCounts (based on featureCounts^39^ version 2.0.1, default settings). Celescope command tools “celescope tag analysis_tag” and “celescope tag split_tag” were used to decode the sample information based on CLindex® sequences.

The primary analyses of the gene-cell expression matrix were performed using Seurat^40^ (V5), encompassing data filtering, normalization, dimension reduction, sample integration, and cell clustering. Only genes expressed in at least three cells were kept, and cells were filtered based on criteria including the number of genes expressed (200 < number of expressed genes < 7000), percentage of mitochondrial transcripts (< 10%), and number of Unique Molecular Identifier (UMI) counts (1000 < UMI counts < 50,000). UMI counts underwent normalization and scaling using the default settings of the NormalizeData and ScaleData functions within the Seurat package. Data from different samples were further integrated using the Harmony^41^ method integrated into the Seurat package, with PCA results serving as template reduction inputs. Following integration, clustering and dimension reduction analyses were conducted using the FindClusters and RunUMAP functions of the Seurat package, respectively, with the clustering resolution set to 0.3. The FindAllMarkers function from Seurat was employed for marker gene identification (min.pct = 0.25, logfc.threshold = 0.25), and *P*-values were adjusted using Bonferroni correction. Significant marker genes were defined as those with adjusted *P*-values less than 0.05.

### Age-related differentially expressed gene analyses

Age-related Differentially Expressed Genes (age-DEGs) were calculated using the Seurat^40^ (V5) function FindMarkers, employing Wilcoxon Rank Sum Test method for statistical testing and utilizing normalized UMI values as input. The log2-transformed fold-change of the average expression (avg_log2FC) between old and young groups was calculated, with a pseudo-value (0.1) incorporated to prevent division by zero. Positive values indicate higher expression in cells from the old mice group. To address multiple comparisons, *P*-values from the Wilcoxon test were adjusted using Benjamini & Hochberg (BH) method. Age-DEGs were defined as the genes with BH-adjusted *P*-values less than 0.05, absolute values of the log2-transformed fold change greater than 1, and a percentage of cells expressed exceeding 10%.

### Gene set enrichment analysis

Gene set enrichment analysis (GSEA) was performed to identify enriched pathways associated with testis aging. Initially, genes detected in each cell type were ranked based on the avg_log2FC values calculated in the age-DEG analyses. These gene rankings were then used as input for the R ClusterProfiler^42^ package to perform GSEA analysis. GSEA of gene ontology (GO) was conducted using the gseGO method from ClusterProfiler. Meanwhile, GSEA for Kyoto Encyclopedia of Genes and Genomes (KEGG) pathways utilized the gseKEGG method from the same package. For the gseGO analysis, the minimum gene set size was set at 30, with a maximum gene set size of 500. Similarly, for the gseKEGG analysis, the minimum gene set size was set at 10, while the maximum gene set size remained at 500. *P*-values for multiple comparisons were adjusted using Benjamini & Hochberg (BH) method, and the significant KEGG or GO terms were defined as those with adjusted *P*-values less than 0.1.

### Age-related alternative splicing analyses

At the outset of our analyses, we partitioned each sample’s associated BAM file, containing genome mapping information, into sub-BAM files according to the cell type annotations. Subsequently, we utilized the rMATS^29^ software to compute alternative splicing (AS) changes between the old and young samples for each cell type, using the GTF format GENCODE annotation file (Release version M25) as a guide for gene structures. Significant AS events were defined as those with FDR less than 0.05 and inclusion level changes greater than 0.2. Moreover, the rmats2sashimiplot tool (version 2.0.4, https://github.com/Xinglab/rmats2sashimiplot) was used to generate Sashimi plots illustrating the AS events.

### RT-PCR and qRT-PCR

Testicular tissues from both young and old mice were collected and placed into 1.5 ml centrifuge tubes, then stored in the refrigerator at − 80 °C. Total RNA extraction from mouse testis was performed using TRIzol Reagent (Cat. No. 15596026, Invitrogen, USA), followed by reverse transcription using HiScript II Reverse Transcriptase Vaz (Cat. No. R312-02, Vazyme, China) to obtain complementary DNA (cDNA) templates. Real-time PCR (qPCR) was conducted using a LightCycler 96 real-time fluorescence quantitative PCR instrument (Roche, Switzerland), with qPCR reaction mixes prepared using ChamQ SYBR qPCR Master Mix (High ROX Premixed) (Cat. No. Q341, Vazyme, China). Each gene studied by qPCR underwent three measurements in at least three biological replicates for each age group. Relative expression values were determined using the 2^−ΔΔCT^ method. Initially, these values were normalized to the expression level of the housekeeping gene Gapdh. Subsequently, the expression value of each sample was normalized to the average expression values of the young group. PCR amplifications were performed using BIO-RAD Thermal cyclers. All primer sequences (Table S15) were synthesized by GenScript (China). The Percent Spliced In (PSI), based on the band intensities, was defined as:

$$PSI=\frac{{I}_{i} }{{I}_{i}+{I}_{e}}$$

where ${I}_{i}$ is the intensity value of the exon-included PCR product band, and ${I}_{e}$ is the intensity value of the exon-excluded PCR product band.

### RNA in situ hybridization

The cDNA templates from both young and old mice were prepared using the method mentioned above, and they were further amplified through PCR (primer sequences in Table S15). The amplified cDNA fragments were then inserted into pGEM®-T Easy Vector Systems (Cat. No. A1360, Promega, USA). Antisense RNA probes labeled with digoxigenin were generated using DIG-RNA Labeling Mix (Cat. No. 11175025910, Roche, Switzerland) following the manufacturer’s instructions.

Testes from both young and old mice were dissected and sectioned into 10 μm slices using a Cryostat (Leica CM1950) at − 23 °C. After that, the slides were warmed to room temperature and dried on a 50 °C slide warmer for 15 min. They were then fixed with 4% PFA for 20 min, followed by digestion with 0.1% Proteinase K Solution (Cat. No. 03115887001, Roche, Germany). After washing with PBS buffer, the slides were fixed again with 4% PFA for 10 min, treated with 0.1 M triethanolamine buffer for 10 min, and prehybridized for 1 h in a humidified hybridization chamber at room temperature.

The digoxigenin-labeled RNA probes were diluted to 1 ng/μL in the hybridization solution, denatured by heating at 85 °C for 5 min followed by ice incubation to prevent reannealing. The slides were incubated with diluted RNA probes (100 μL) in the humidified hybridization chamber at 70 °C overnight. Subsequently, the slides were further washed with SSC buffer to remove excess probes and hybridization buffer before being incubated in 1% blocking buffer (Cat. No. 11096176001, Roche, Switzerland) at room temperature for 1 h. Next, the slides were incubated at 4 °C overnight with alkaline phosphatase-conjugated anti-DIG antibody diluted in blocking buffer (1:4000, Cat. No. 11093274910, Roche, Switzerland), followed by treatment with AP-substrate NBT/BCIP solution (Cat. No. 11681451001, Roche, Switzerland). Brightfield images of the sections were obtained using a Nikon Eclipse Ni-U microscope. For negative controls, sense probes were employed alongside the corresponding gene's antisense probe in our RNA in situ hybridization experiments.

### Immunofluorescence staining

Two types of methods were used for tissue preservation in the immunofluorescence (IF) staining experiments: the first one was based on paraffin wax embedding and the other was based on cryopreservation.

For the IF experiments based on paraffin wax embedding, mice underwent transcardial perfusion with PBS followed by 4% paraformaldehyde (PFA) before testis dissection. The dissected testicular tissues were first fixed in modified Davidson’s fluid (mDF) at 4 °C, then transferred to 70% ethanol solution for 2 h at room temperature. After that, the tissues were treated with a graded ethanol series (80–95%) at room temperature for 1 h, followed by dehydration with 100% ethanol twice at room temperature for 30 min. The dehydrated tissues were transparentized with xylene and embedded in paraffin wax. Sections of 5 μm thickness were cut using a microtome (Leica RM2235) and stored at − 20℃ until further processing. Prior to staining, the slides were warmed to room temperature and dried on a 37 °C slide warmer for 2 h. The paraffin slides were deparaffinized in xylene twice for 15 min each. Subsequently, the slides were dehydrated with 100% ethanol and 95% ethanol respectively at room temperature for 5 min, then rehydrated with 70–80% ethanol at room temperature for 3 min, which were further washed by 1X PBS for three times (5 min each). Next, the slides were incubated in pre-warmed antigen retrieval solution at 100 °C for 25 min, followed by washing with 1X PBS three times for 5 min each. The slides were then treated with blocking solution (3% sheep serum, 0.1% BSA, and 0.2% Triton-100) at room temperature for 1 h. After blocking, the slides were incubated with primary antibody (1:50) overnight at 4 °C, followed by three washes with 1X PBS for 5 min each. Next, the slides were incubated with secondary antibody (1:400) diluted in blocking solution for 1 h, followed by washing with 1X PBS. Finally, the slides were mounted with Antifade Mounting Medium containing DAPI (Cat.No. P0131, Beyotime).

For the IF experiments based on cryopreservation, mice were euthanized with a lethal dose of anesthesia, and testicular tissue was promptly harvested. The tissues were fixed in 4% PFA for 48 h, followed by dehydration in sucrose gradients (10% and 30%) at 4 °C. Subsequently, the testes were embedded in optimal cutting temperature (OCT) compound and stored at − 80 °C until further processing. The OCT-embedded testes were sectioned into 10 μm slices using a Cryostat (Leica CM1950) and stored at − 20 °C. Prior to staining, the sections were allowed to reach room temperature and dried on a 37 °C slide warmer for 1 h. The slides were then washed three times with 1X phosphate-buffered saline (PBS) for 5 min each. To minimize non-specific binding, the slides were incubated in blocking solution consisting of 3% sheep serum, 0.1% bovine serum albumin (BSA), and 0.2% Triton X-100 for 1 h at room temperature. Following blocking, the slides were incubated with the primary antibody (diluted 1:100) overnight at 4 °C, followed by eight washes with 1X PBS for 5 min each. After primary antibody incubation, the slides were incubated with secondary antibody (diluted 1:500 in blocking solution) for 1 h at room temperature. Following another round of washing with 1X PBS, the slides were mounted using Antifade Mounting Medium containing 4',6-diamidino-2-phenylindole (DAPI) (#No.P0131, Beyotime) to visualize cell nuclei.

Primary antibodies used were rabbit anti-ACTA2 (1:50 for paraffin/1:100 for cryopreservation, Cat.No. 14395-1-AP, proteintech) and rat anti-CD34 (1:50 for paraffin/1:100 for cryopreservation, Cat.No. sc-18917, Santa Cruz). Secondary antibodies were Cy3-conjugated AffiniPure Goat Anti-Rat IgG (1:400 for paraffin/1:500 for cryopreservation, Cat.No. A0507, Beyotime) and Alexa Fluor 488-conjugated AffiniPure Goat Anti-Rabbit IgG(H + L) (1:400 for paraffin/1:500 for cryopreservation, Cat.No. 111-545-003, Jackson). Slides stained only with secondary antibody were used as negative controls for nonspecific signals.

### Transmission electron microscopy (TEM)

Upon dissection, testicular tissues from both young and old mice were promptly treated with electron microscope fixation solution (Cat.No.G1102, Servicebio, China). The testis specimens were cut into small pieces (1 mm^3^) and fixed with 1% OsO4 (Cat. No. 18456, Ted Pella, USA) in 0.1 M PB (pH 7.4) for 2 h at room temperature in a dark environment. After removal of OsO4, the tissues were rinsed in 0.1 M PB (pH 7.4) three times (15 min each), and then dehydrated with progressively higher concentrations of ethyl alcohol (30%, 50%, 70%, 80%, 95%, 100%, 20 min each) (Cat.No.100092183, Sinopharm Chemical Reagent, China) followed by treatment with acetone (Cat. No. 10000418, Sinopharm Chemical Reagent, China) for 15 min. After that, the tissues were embedded in EMBed 812 (Cat. No. 90529-77-4, SPI Supplies, USA) and sectioned into slices of 70 nm thickness using a Leica EM UC7 Ultramicrotome (Leica, Germany), which were then mounted on copper-coated grids. The sections were stained with uranium acetate saturated alcohol solution (2%, avoid light) for 8 min, followed by triple rinsing with 70% ethanol and three additional rinses in ultra-pure water. Subsequently, the sections were stained with 2.6% lead citrate to avoid CO2 staining for 8 min, followed by three rinses with ultra-pure water. The cuprum grids were examined using Transmission Electron Microscopy (TEM) (HT7800, HITACHI, Japan).

### Quantification and statistical analysis

All statistical details, including the applied statistical method, sample size, and significance assessed by *P*-values, were clearly delineated in the figure legends. The results from replicate experiments, depicted in bar plots, were presented as mean ± standard error of the mean (SEM). To study mitochondrial changes based on TEM experiments, the number of mitochondria within telocytes was normalized to the standard telopode length (20 μm).

For quantitative analysis of the in situ hybridization (ISH) results, images were imported into ImageJ software (version 1.54d) and converted to RGB stacks. The RGB stack image with the strongest correlation to positive staining signals was selected. Manual thresholding was then applied to identify pixels displaying positive staining signals within the range of 0–129. Subsequently, the fraction of the area displaying positive staining signals was quantified using ImageJ.

All statistical analyses for experimental results were performed using GraphPad Prism (version 8.4.3), with *P*-values calculated using a two-sided Student’s t-test.

### Supplementary Information

Supplementary Information 1.

Supplementary Information 2.

Supplementary Information 3.

Supplementary Information 4.

Supplementary Information 5.

Supplementary Information 6.

Supplementary Information 7.

Supplementary Information 8.

Supplementary Information 9.

Supplementary Information 10.

## Data Availability

The scRNA-seq gene expression matrix files and Seurat object file generated in this study can be found on figshare (10.6084/m9.figshare.25034684). The code used for the scRNA-seq data analysis is available on github (https://github.com/gangcai/TestisAgingScRNASeq). The raw sequencing data and the reads alignment information (BAM files) reported in this paper have been deposited in the Genome Sequence Archive (GSA) of the China National Center for Bioinformation (CNCB) (GSA: CRA014599), which will be publicly accessible at https://ngdc.cncb.ac.cn/gsa. The primer sequences for the PCR/qPCR and ISH experiments are provided in the supplementary Table S15.
