# Supplementary material for: Single-cell transcriptome profiling highlights the importance of telocyte, kallikrein genes, and alternative splicing in mouse testes aging
Source: Sci Rep. 2024 Jun 26;14:14795. doi: 10.1038/s41598-024-65710-0 (PMC11208613; doi:10.1038/s41598-024-65710-0)
Supplement: Supplementary file 1 — Supplementary Information 1. [file 41598_2024_65710_MOESM1_ESM.pdf]

SUPPLEMENTARY DATA

Supplementary Figures:

Figure S1: ScRNA-seq data basic statistical metrics.
Figure S2: Telocyte subpopulation analysis.
Figure S3: Expression of Kallikreins genes in mouse testes.
Figure S4: Klk1b22 in situ hybridization based on sense probe.
Figure S5: Age-related mitochondrial gene changes in testicular telocytes.
Figure S6: Immunofluorescence staining of CD34 and ACTA2.
Figure S7: Raw data of the agarose gel electrophoresis validation of the RT-PCR products of 4930555F03Rik transcripts in the young and old mouse testes.

Supplementary Tables:

Table S1: Animal information for scRNA-seq experiments.
Table S2: Source of the known marker genes.
Table S3: List of identified marker genes for each cell type.
Table S4: Marker genes of telocyte subpopulations
Table S5: List of age-DEGs.
Table S6: KEGG GSEA results for age-DEGs.
Table S7: Age-related cell population proportion changes.
Table S8: Age-related expression changes of mitochondrial genes in telocytes.
Table S9: List of significant SE events.
Table S10: List of significant A5SS events.
Table S11: List of significant A3SS events.
Table S12: List of significant MXE events.
Table S13: List of significant RI events.
Table S14: Animal information for validation experiments.
Table S15: Primer sequences.

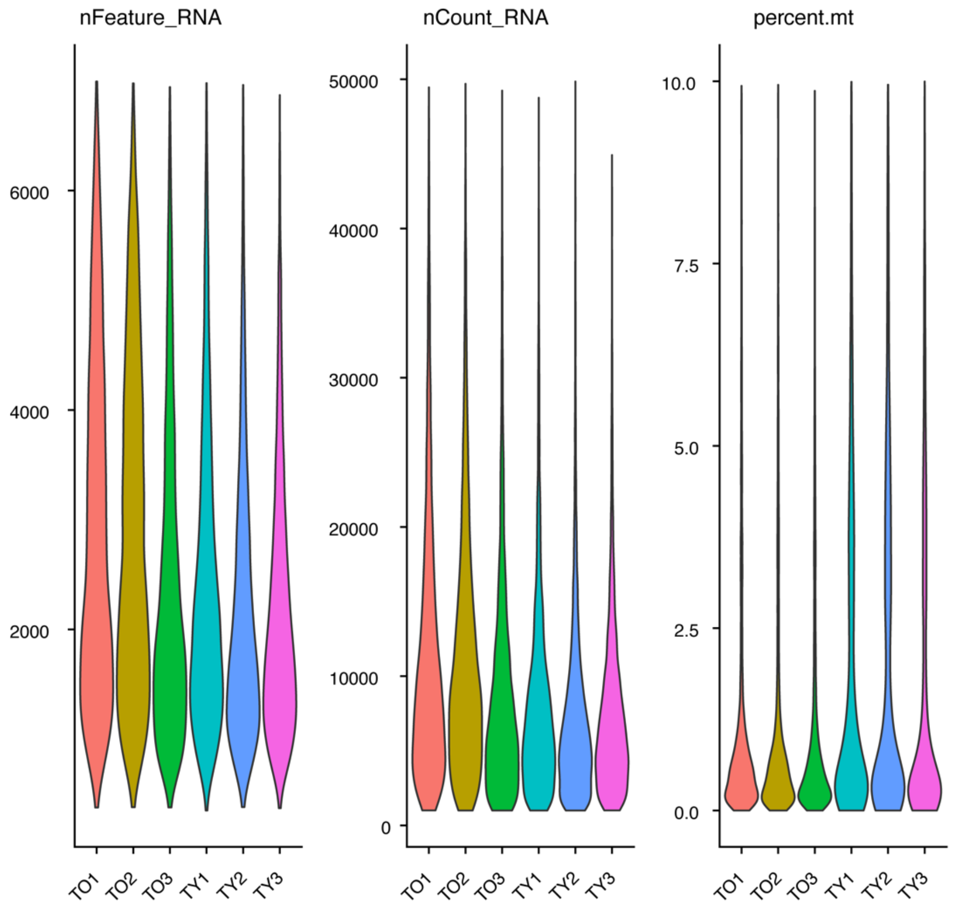

Figure S1: ScRNA-seq data basic statistical metrics.

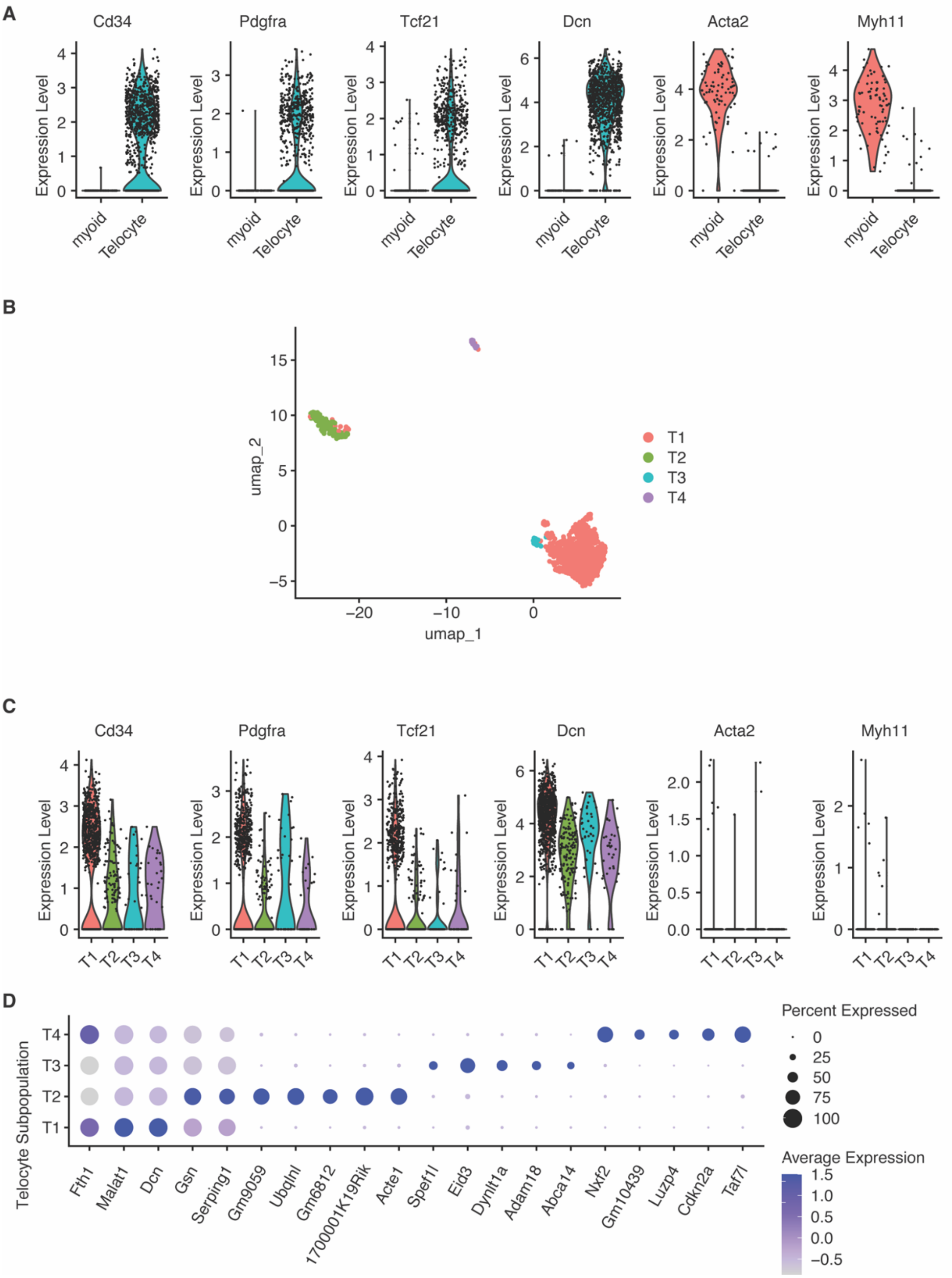

Figure S2: Telocyte subpopulation analysis. (A) Comparison of the expression of the marker genes of myoid cells (Acta2, Myh11) and telocytes (Cd34, Pdgfra, Tcf21, Dcn). (B) UMAP representation of the four telocyte subpopulations (T1, T2, T3, T4). (C) Violin plots of the marker genes of myoid cells and telocytes in the four telocyte subpopulations. (D) Dot plot of the expression of the top five marker genes of telocyte subpopulations.

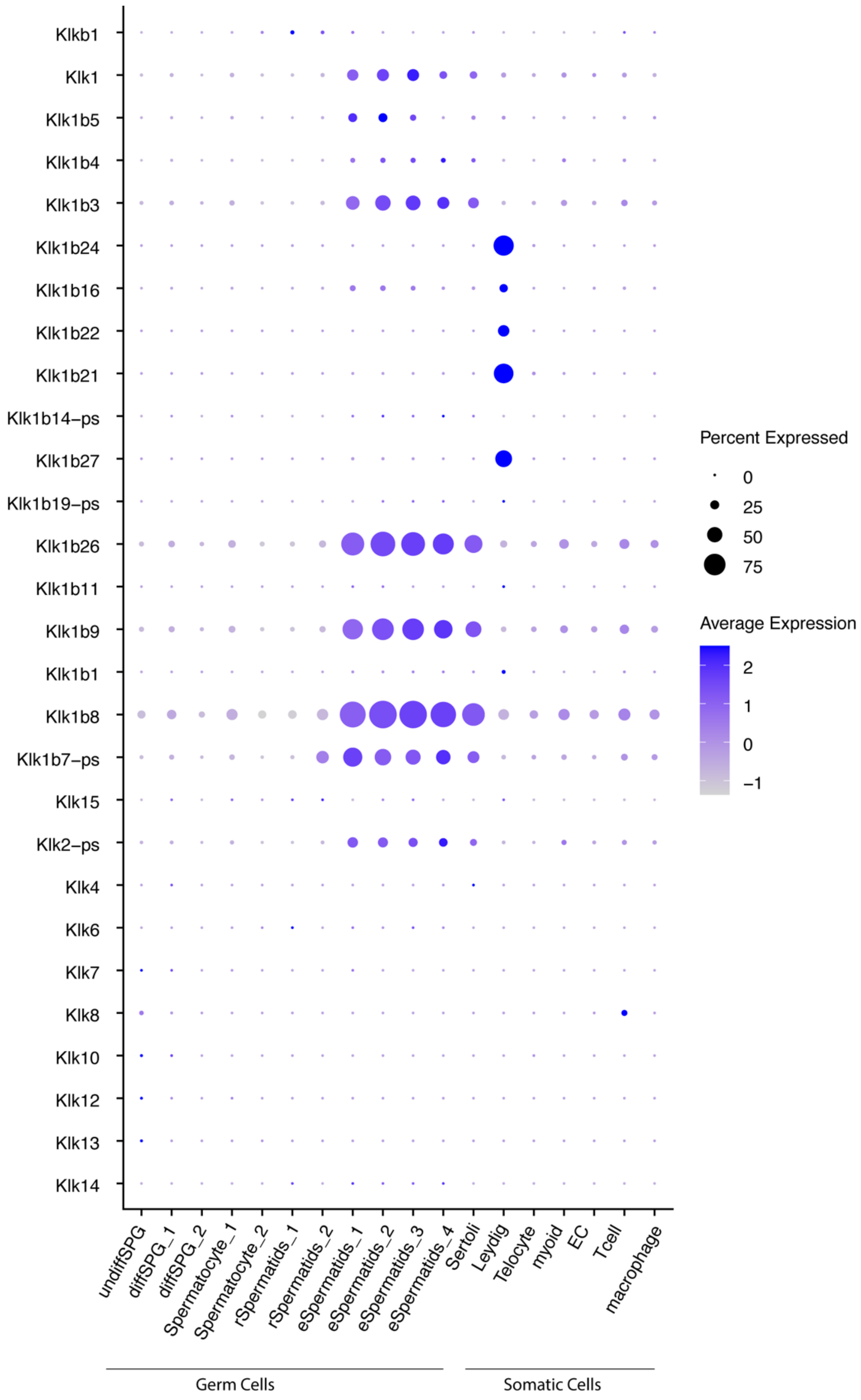

Figure S3: Expression of Kallikreins genes in mouse testes.

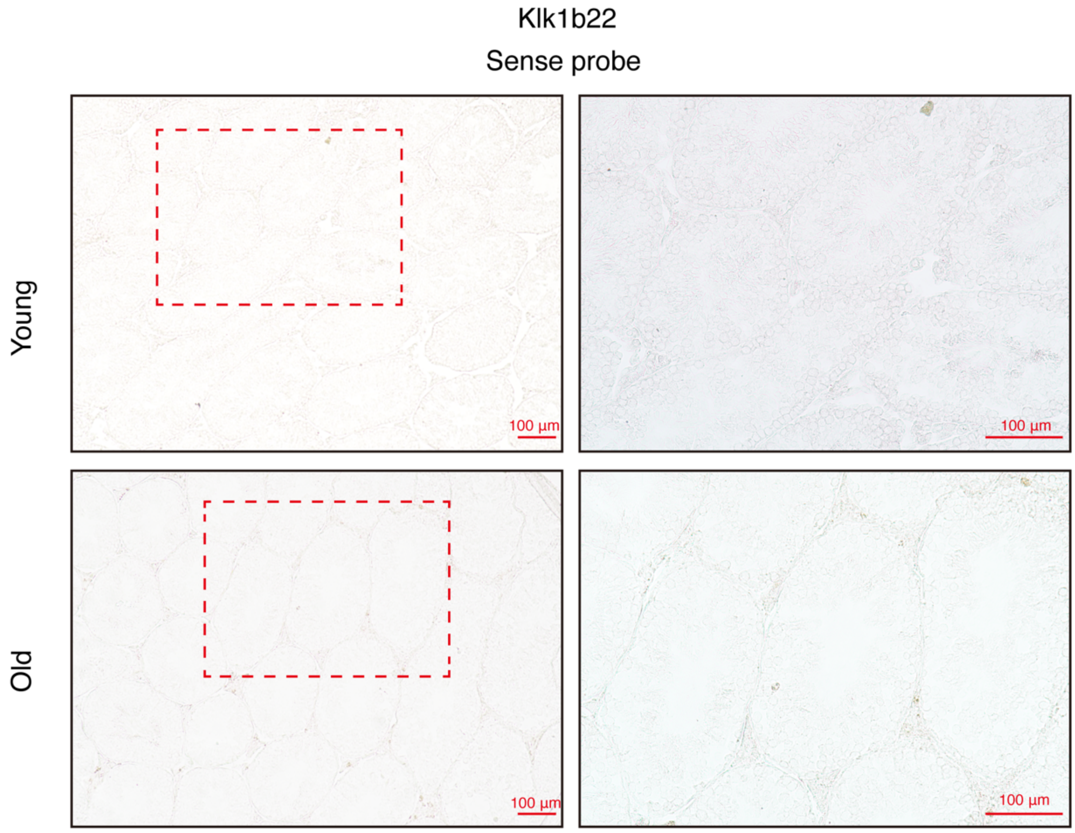

Figure S4: Klk1b22 in situ hybridization based on sense probe.

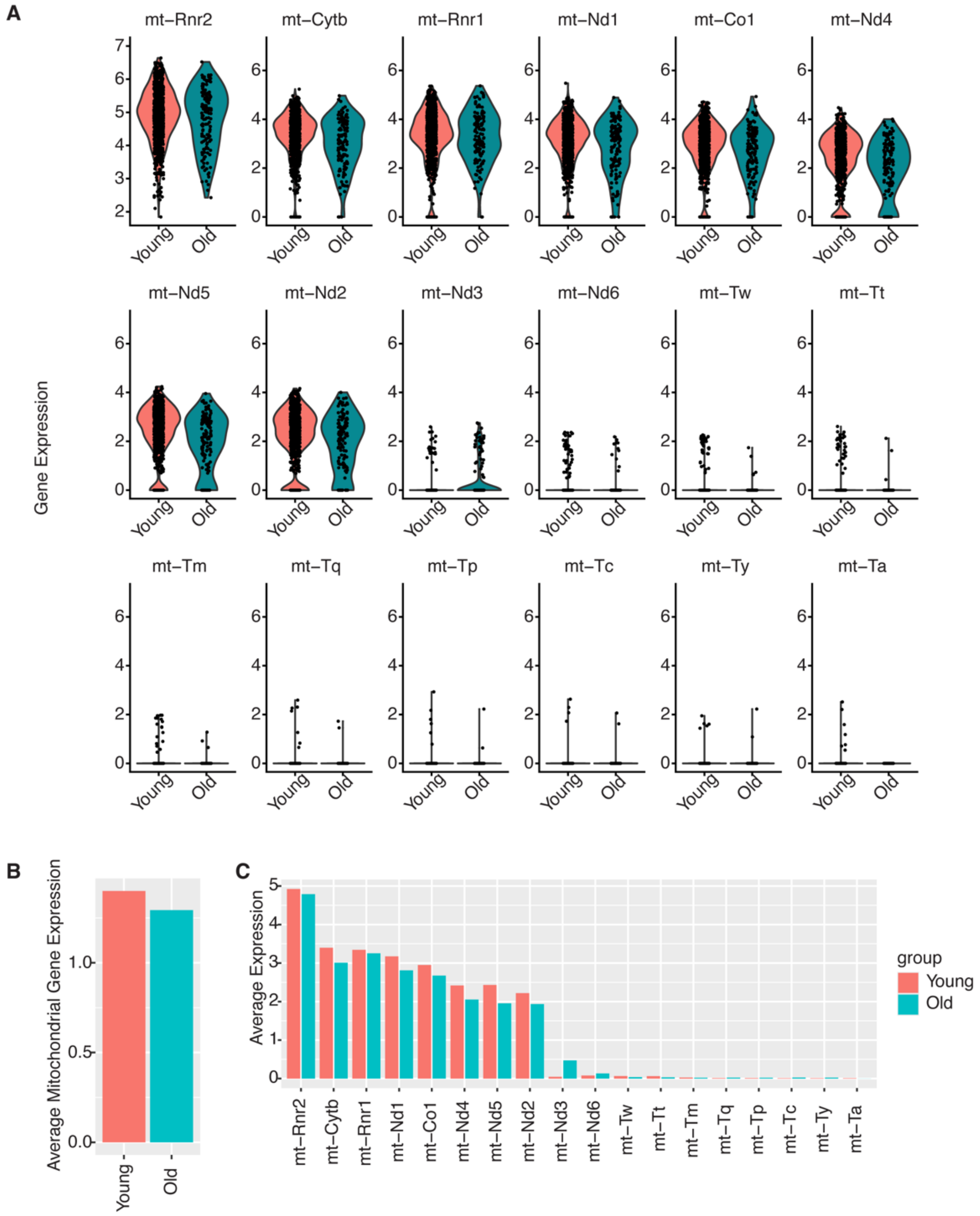

Figure S5: Age-related mitochondrial gene changes in testicular telocytes. (A) Violin plots of all the detected mitochondrial genes in the telocytes of young and old mouse testes. (B) Global average expression of the detected mitochondrial genes in the telocytes of young and old mouse testes. (C) Average expression of each mitochondrial gene detected in the telocytes of young and old mouse testes.

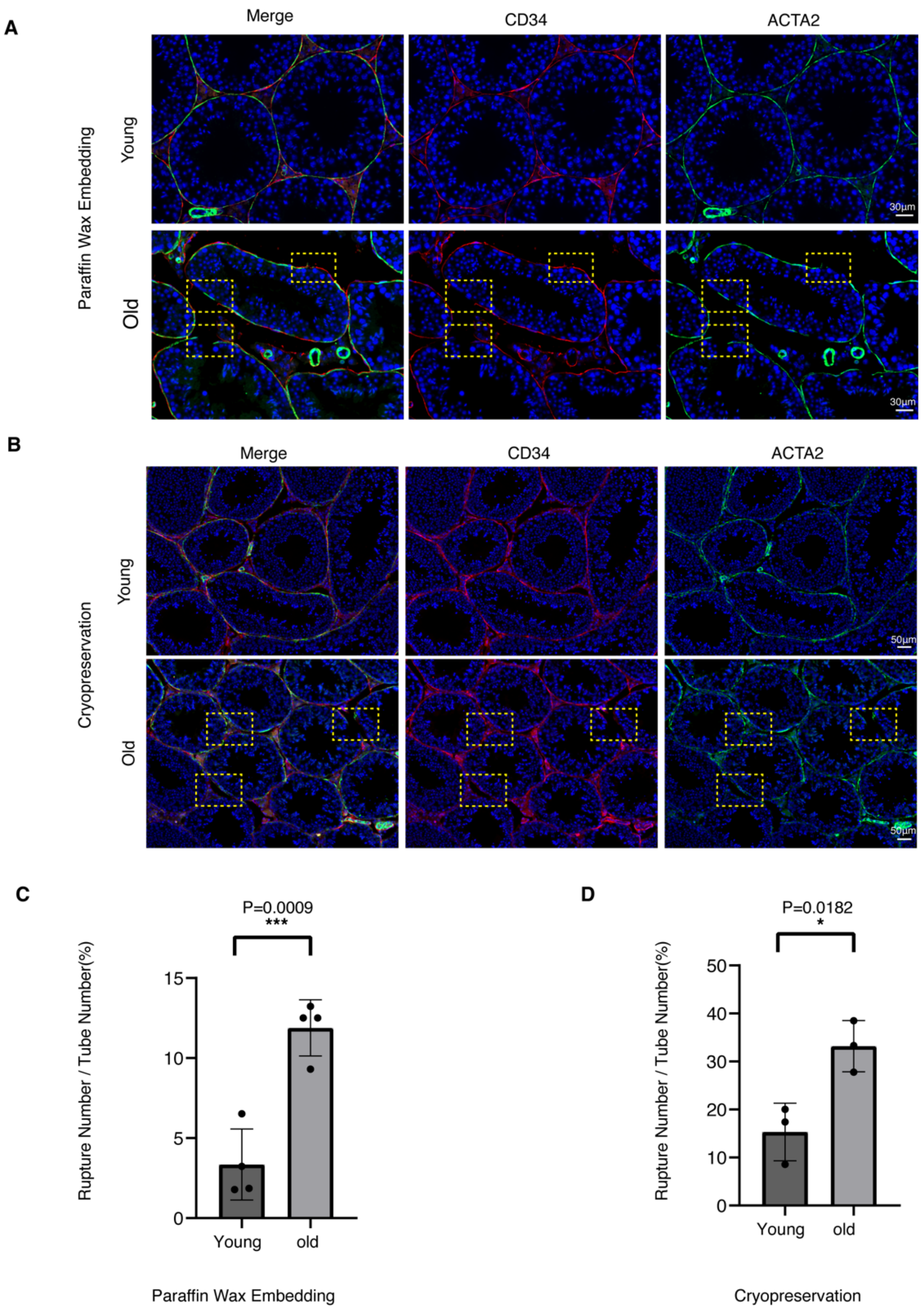

Figure S6: Immunofluorescence staining of CD34 and ACTA2. (A) Immunofluorescence staining of CD34 and ACTA2 based on paraffin embedding. Scale bar=30μm. (B) Immunofluorescence staining of CD34 and ACTA2 based on cryopreservation. Scale bar=50μm. PMCs: ACTA2 positive (green); Telocytes: CD34 positive (red). The damaged telocyte connections are highlighted by the box. (C, D) Comparison of telocyte rupture sites between old and young mice based on paraffin embedding (C) or cryopreservation (D). Error bars represent the SEM and *P*-values were calculated using two-sided Student's unpaired *t*-tests. *** $P < 0.001$, Young mice (n=4), Old mice (n=4).

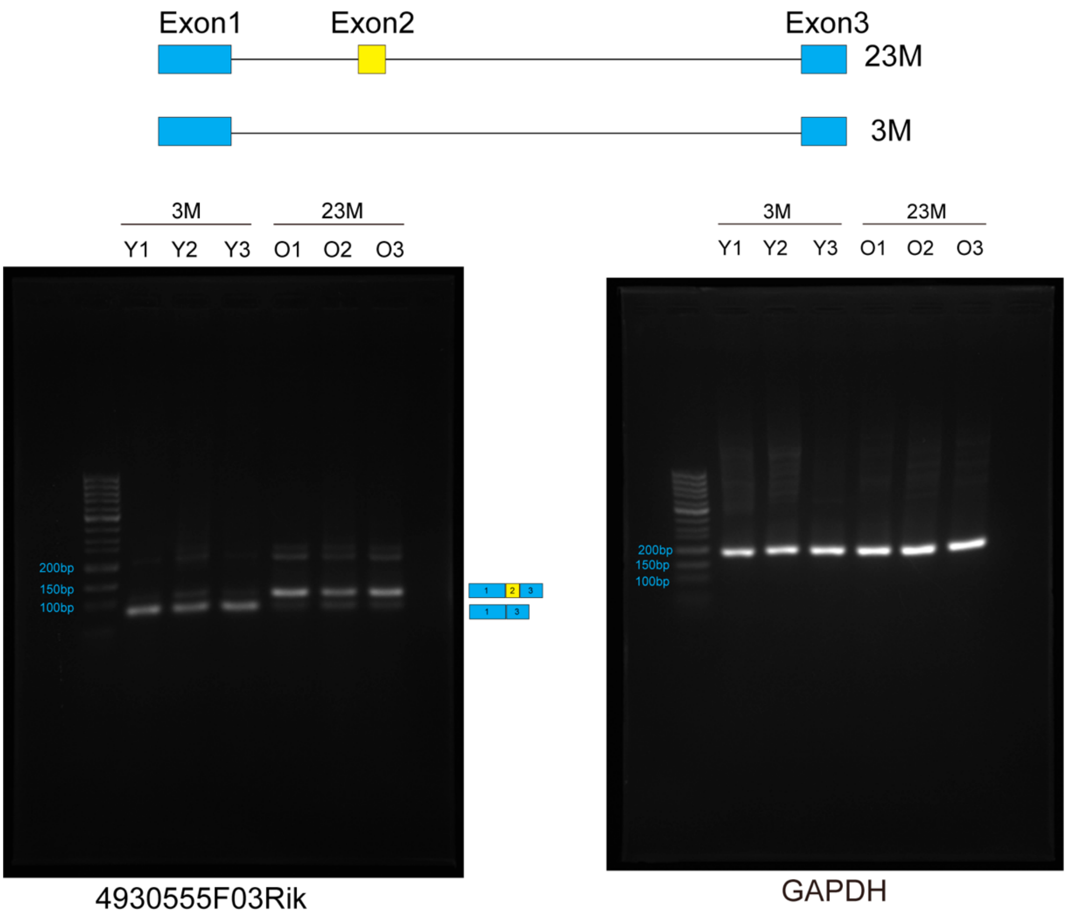

Figure S7: Raw data of the agarose gel electrophoresis validation of the RT-PCR products of 4930555F03Rik transcripts in the young and old mouse testes.

Sample_ID	Age	Sex	Strain	Date of Birth	Experiment_Date
TY1	3M	male	C57BL/6	2021/12/16	2022/3/30
TY2	3M	male	C57BL/6	2021/12/16	2022/3/30
TY3	3M	male	C57BL/6	2021/12/16	2022/3/30
TO1	23M	male	C57BL/6	2020/4/15	2022/3/30
TO2	23M	male	C57BL/6	2020/4/15	2022/3/30
TO3	23M	male	C57BL/6	2020/4/15	2022/3/30

Table S1: Animal information for scRNA-seq experiments.

CellType	MarkerGene	PMID
undiffSPG	Zbtb16	30146481
undiffSPG	Lin28a	31237565
diffSPG	Kit	30146481
diffSPG	Stra8	30146481
Spermatocytes	Piwil1	30146481
Spermatocytes	Pttg1	30146481
Round_spermatids	Acrv1	30893341
Round_spermatids	Tex21	30890697
Round_spermatids	Spaca1	34125190
Round_spermatids	Spaca3	34125190
Elongating_spermatids	Cby3	31237565
Elongating_spermatids	Prm1	30146481
Elongating_spermatids	Prm2	30146481
Elongating_spermatids	Tnp1	30146481
Elongating_spermatids	Tnp2	30146481
Sertoli	Cst12	31237565
Sertoli	Cldn11	30890697
Sertoli	Rhox8	30146481
EC	Pecam1	30576429
EC	Flt1	31964773
Leydig	Star	30146481
Leydig	Cyp11a1	30146481
Macrophage	Cd68	31237565
Macrophage	Csf1r	31237565
Telocyte	Pdgfra	31237565
Telocyte	Dcn	31237565
Telocyte	Cd34	31237565
Telocyte	Tcf21	31237565
T-cell	Cd2	31237565
T-cell	Cd3e	31237565
Myoid	Acta2	30146481
Myoid	Myh11	30146481

Table S2: Source of the known marker genes.

CellType	TO1	TO2	TO3	TY1	TY2	TY3	log2FC	pvalue
myoid	0.00049938	0.00080699	0.00042499	0.00330033	0.00273696	0.00347423	-2.4577719	0.00186963
Leydig	0.01248439	0.00968393	0.00864145	0.02855503	0.02837799	0.02301679	-1.3757066	0.00306534
diffSPG_1	0.08227216	0.06415602	0.07593144	0.13129574	0.13814463	0.11624204	-0.7945185	0.00327656
diffSPG_2	0.0258427	0.02232683	0.01954951	0.05467068	0.05934889	0.04371743	-1.2198877	0.01355781
Telocyte	0.00474407	0.00659045	0.00679983	0.04161286	0.05214636	0.03546613	-2.833092	0.0155329
rSpermatids_2	0.10461923	0.11284465	0.08244794	0.058258	0.04393547	0.04675738	1.00970204	0.01697372
Sertoli	0.01672909	0.01412239	0.0161496	0.01951499	0.01973495	0.02041112	-0.3440957	0.0237273
Spermatocyte_1	0.09026217	0.09119032	0.0772064	0.06457167	0.04263901	0.03821656	0.83075322	0.025107
undiffSPG	0.01635456	0.01613988	0.01770789	0.02826804	0.03457217	0.02605675	-0.8243799	0.03304143
EC	0.00649189	0.00766644	0.00878311	0.01018798	0.01699798	0.01389693	-0.8405816	0.07921462
Spermatocyte_2	0.12684145	0.10773369	0.0560986	0.04390874	0.02679343	0.03445281	1.46688358	0.09259863
rSpermatids_1	0.10224719	0.10827169	0.0626151	0.06385421	0.04436762	0.04588882	0.82564221	0.09282075
eSpermatids_1	0.03171036	0.04707465	0.04561553	0.02367628	0.03053875	0.03112334	0.54372631	0.09968186
eSpermatids_4	0.16941323	0.1604573	0.21277801	0.19371502	0.22082973	0.24203822	-0.2749592	0.15161429
macrophage	0.00873908	0.01977135	0.01232469	0.01621466	0.02607318	0.0208454	-0.6285893	0.16170882
eSpermatids_2	0.06404494	0.07182246	0.09080606	0.08250825	0.0707289	0.08714534	-0.0847163	0.6548136
eSpermatids_	0.134207	0.134633	0.197903	0.130291	0.135839	0.166039	0.111031	0.662404

3	24	49	39	29	82	37	31	57
Tcell	0.00249688	0.00470746	0.00821646	0.00559621	0.00619418	0.00521135	-0.1408044	0.78316781

Table S7: Age-related cell population proportion changes.

genes	cmp_info	cluster.info	p_val	avg_log2FC (old vs young)	pct.1	pct.2	p_val_adj	average_expression
mt-Rnr2	Old_vs_Young	Telocyte	0.27309422	-0.07294772	1	1	1	4.90637709
mt-Cytb	Old_vs_Young	Telocyte	4.68E-05	-0.344459386	0.978	0.988	1	3.34603989
mt-Rnr1	Old_vs_Young	Telocyte	0.16366339	-0.023147924	0.985	0.968	1	3.330216665
mt-Nd1	Old_vs_Young	Telocyte	0.00133514	-0.278481748	0.963	0.978	1	3.125622413
mt-Co1	Old_vs_Young	Telocyte	0.00130291	-0.260492012	0.963	0.964	1	2.913738525
mt-Nd4	Old_vs_Young	Telocyte	7.00E-05	-0.434721853	0.844	0.891	1	2.370955083
mt-Nd5	Old_vs_Young	Telocyte	4.89E-07	-0.592725193	0.83	0.894	0.016936614	2.368761073
mt-Nd2	Old_vs_Young	Telocyte	0.00410771	-0.314936937	0.837	0.851	1	2.181178743
mt-Nd3	Old_vs_Young	Telocyte	6.00E-30	2.784155682	0.274	0.027	2.08E-25	0.10218527

Table S8: Age-related expression changes of mitochondrial genes in telocytes.

Experiments	Samples (Young)	Samples (Old)	Mouse Strain	Mouse Sex
RT-PCR	Y1：3M，Y2：3M，Y3：3M	O1：23M，O2：23M，O3：23M	C57BL/6	Male
qRT-PCR (Fmn1)	Y1：3M，Y2：3M，Y3：3M，Y4：3M	O1：23M，O2：23M，O3：23M，O4：21M	C57BL/6	Male
qRT-PCR (Klk1b22, Klk1b24)	Y1：3M，Y2：3M，Y3：3M，Y4：3M	O1：23M，O2：23M，O3：23M	C57BL/6	Male
Transmission electron microscopy	Y1：3M，Y2：3M	O1：30M，O2：27M	C57BL/6	Male
RNA in situ hybridization	Y1：3M，Y2：3M，Y3：3M，Y4：3M	O1：23M，O2：24M，O3：23M，O4：23M	C57BL/6	Male
Immunofluorescence Staining (Paraffin Wax)	Y1：3M，Y2：3M，Y3：3M，Y4：3M	O1：21M，O2：23M，O3：29M，O4：29M	C57BL/6	Male
Immunofluorescence Staining (Cryopreservation)	Y1：3M，Y2：3M，Y3：3M	O1：24M，O2：23M，O3：23M	C57BL/6	Male

Table S14: Animal information for the validation experiments.

Primers	Primer Sequence (5'- 3')
**Housekeeping gene**	
GAPDH-Forward Primer	AACGACCCCTTCATTGAC
GAPDH-Reverse Primer	TCCACGACATACTCAGCAC
**ISH**	
Klk1b22-Forward Primer	GATTGATGCTGCACCTCCTG
Klk1b22-Reverse Primer	GCTTGATGGACACACACTGG
**RT-PCR**	
4930555F03Rik-Forward Primer	TCCTGAGGAAGAAGGTGTGGA
4930555F03Rik-Reverse Primer	GCTTGCATGCATCTCTTTCTTTC
**RT-qPCR**	
Klk1b22-Forward Primer	TTGGTCAGCAAAAGCTTCCC
Klk1b22-Reverse Primer	CTGTGATGTCAGCAGGCTTG
Klk1b24-Forward Primer	CAATGACCTGATGCTGCTCC
Klk1b24-Reverse Primer	TTTCGTGGGTGTAATGCTGC
Fmn1-Forward Primer	TGACACCTTGGAGCCATCAT
Fmn1-Reverse Primer	TGATCTTCTCTGGCCCCAAG

Table S15: Primer sequences.
